# Impaired configural processing for other-race faces revealed by the Thatcher illusion 

**DOI:** 10.1177/20416695261480594

**Published:** 2026-09-07

**Authors:** Sandra Utz, Claus-Christian Carbon

**Affiliations:** 1 Department of General Psychology and Methodology, 14310 University of Bamberg, Bamberg, Germany; 2 Forschungsgruppe EPÆG (Ergonomics, Psychological Aesthetics, Gestalt), Bamberg, Germany; 3 Bamberg Graduate School of Affective and Cognitive Sciences (BaGrACS), University of Bamberg, Bamberg, Germany

**Keywords:** faces, Thatcher illusion, own-race bias, morphological group, configural processing, featural processing

## Abstract

The Thatcher illusion, where participants perceive upright faces with inverted eyes and mouth as grotesque but are markedly impaired in detecting grotesqueness when the entire faces are inverted, is commonly interpreted as reflecting disrupted configural and/or holistic processing in inverted faces. Previous research has suggested that other-race faces may be processed with a comparatively reduced reliance on configural (and more featural) information than own-race faces. The own-race bias refers to the tendency to recognise faces from a more familiar morphological group – mostly the own ‘race’ – faster and with higher accuracy than faces from less familiar groups. The present study tried to identify differences in processing own- and other-race faces (Thatcherised or not) across seven orientations ranging from 0° to 180° in 30° steps. For own-race faces, reaction times (RTs) to Thatcherised stimuli descriptively followed a sigmoidal function with a marked increase around 120° rotation, reflecting a typical reduced efficiency of configural processing once faces were rotated by more than 90°. For other-race faces, RTs increased more gradually and approximately linearly across orientations, consistent with reliance on featural processing. Accuracy was lowest for Thatcherised other-race faces with a steep decline starting between 30° and 60° of rotation. Overall, the results suggest a processing advantage for own-race faces that may rely more strongly on configural processing, whereas other-race faces appear to be processed with comparatively greater reliance on feature-based strategies.

## How to Cite this Article

Utz, S., & Carbon, C.-C. (2026). Impaired configural processing for other-race faces revealed by the Thatcher illusion. *i-Perception*, *17*(5), 1-17. https://doi.org/10.1177/20416695261480594

## Introduction

There are few classes of stimuli humans encounter as frequently in everyday life as faces. Accordingly, most adults (with a few exceptions, e.g., people with prosopagnosia) develop a high level of expertise in facial processing. This expertise enables rapid and reliable face recognition even under suboptimal viewing conditions, such as poor illumination, changes in hairstyle, large viewing distances or long interruptions in contact (Maurer et al., 2002), particularly for familiar faces, although different levels of familiarity may rely on qualitatively similar representational mechanisms (Carbon, 2008; Wiese et al., 2022). Faces convey highly relevant social information, including identity, age, gender, emotional state (Armann, 2011; Simion & Di Giorgio, 2015) or the mental state of an interaction partner (Schmidtmann et al., 2020). Unlike many other object classes, faces share a highly similar structure or configural base arrangement, the so-called ‘first-order relationship’ (Maurer et al., 2002), consisting of a nose located between two horizontally aligned eyes above a mouth. However, identifying an individual face requires processing more subtle spatial relationships among facial features, commonly referred to as ‘second-order relationships’ (Armann, 2011; Mondloch et al., 2002). This expertise, however, develops gradually from infancy through adolescence into adulthood and is also subject to qualitative changes (Joseph et al., 2015; Maurer et al., 2002) and is not fully developed before the 15^th^ up to 21^st^ year of life (Carbon et al., 2013; Uhlhaas et al., 2009). Importantly, developmental changes in face expertise appear to involve an increasing reliance on configural and/or holistic processing strategies, whereas children rely more strongly on piecemeal processing, the so-called featural or non-configural processing (e.g., Bülthoff & Ruppertsberg, 2006; Carbon et al., 2013; Joseph et al., 2015; Mondloch et al., 2002). Despite extensive research, there is still disagreement about a uniform definition of configural face processing. In an overview article, Maurer et al. (2002) postulated three partially overlapping types of configural face processing: (1) Sensitivity to first-order features allows rapid identification of stimuli as faces based on the presence of eyes, nose, and mouth and their relative position to one another. (2) Special attention to first-order relations is sufficient to *detect* faces, but to *recognise* individual faces, the processing of second-order relations containing the spatial arrangement of the internal facial features such as interocular distance, position of the nose, and nose–mouth distance is particularly helpful (Maurer et al., 2002). Adults are quite good at recognising and differentiating faces where the second-order relationship between features has been minimally changed (e.g., the distance between the eyes, the position of the mouth and the nose changed by a maximum of three pixels; Freire et al., 2000). However, this performance declines when a memory task is added, and more recent studies suggest that this ability may be less robust than previously assumed (e.g., Burton et al., 2015). This capability seems to be corrupted or at least impaired in the case of congenital prosopagnosia (cPA) (Grüter et al., 2008), which leads to pronounced face recognition problems (Behrmann & Avidan, 2005; Lobmaier et al., 2010) and qualitatively different face processing strategies (Carbon et al., 2010; Carbon et al., 2007). (3) ‘Holistic processing’ which refers to the processing of a face as an integrated Gestalt. The distinction between second-order and holistic processing remains debated. Holistic processing has been investigated using several paradigms, including the part-whole paradigm employed by Tanaka and Farah (1993) and extended by Leder and Carbon (2005), the composite face paradigm (e.g., de Heering et al., 2008; Hole et al., 1999; Young et al., 2013) or the Mooney face paradigm (Carbon et al., 2013; McKone, 2009; Mooney, 1957).

Strong evidence for expertise-based configural processing, especially for processing second-order relations, is the face inversion effect (Yin, 1969), showing that the recognition performance for faces deteriorates substantially when faces are presented upside-down – in an ‘inverted’ orientation (e.g., Farah et al., 1998; Leder & Carbon, 2006; Rhodes et al., 1993; Rossion, 2008). Compared to faces, most objects are less affected by inversion because they can often be classified on the basis of a limited number of distinctive local features (e.g., the specific front lights or the grill of a car) (Belke et al., 2010; Joseph et al., 2015). However, inversion does not selectively impair only one aspect of face perception. Rather, studies suggest that inversion affects multiple aspects of processing simultaneously, including first-order relations (e.g., Maurer et al., 2002), second-order relations (e.g., Freire et al., 2000; Leder & Carbon, 2006) and holistic/configural processing (e.g., Carey & Diamond, 1994). Consequently, inversion paradigms should be interpreted cautiously with regard to claims about isolated perceptual mechanisms.

One widely used paradigm demonstrating inversion-related impairments in face processing is the Thatcher illusion (Thompson, 1980), where participants perceive upright faces – illustrated in Thompson's demonstration with a photograph of former Prime Minister Margaret Thatcher – as grotesque when the eyes and mouth are inverted, but their ability to detect this grotesqueness is markedly reduced when the entire face is inverted. The Thatcher illusion is interpreted as reflecting disrupted configural and/or holistic processing mechanisms for inverted faces (Lewis & Johnston, 1997). Carbon et al. (2007) compared the performance of adults to people with cPA (a group known to have a certain dysfunction with regard to face expertise processing) with age- and sex-matched controls using a modified Thatcher illusion paradigm. Thatcherised and normal faces were rotated in seven steps between 0° [upright] and 180° [inverted], that is, 0°, 30°, 60°, 90°, 120°, 150° and 180°. For control participants, reaction times (RTs) and accuracy showed an abrupt transition around 90° rotation, whereas individuals with cPA showed more gradual, approximately linear response profiles. These dissociating trends were interpreted as a bimodal processing of featural plus configural information for controls versus a much simpler processing for prosopagnosics who used featural processing only. Carbon et al. interpreted these findings as reflecting a stronger reliance on configural processing in controls and a comparatively greater reliance on feature-based processing in cPA. Featural processing is much less vulnerable to inversion (Leder & Carbon, 2006). Importantly, however, such interpretations should be considered cautiously, as inversion likely affects multiple components of face processing simultaneously.

Not only inversion but also familiarity with different morphological groups appears to affect face-processing efficiency: The so-called ‘other-race^1^ effect’ or ‘own-race bias’ describes the robust finding that individuals are generally less accurate and slower when recognising faces from less familiar morphological groups (e.g., Malpass & Kravitz, 1969; Meissner & Brigham, 2001; Tanaka et al., 2004; Young et al., 2012). In the face literature, morphological groups are mostly qualified by ‘race’ – the latter term we will use to be compatible with the terminology of the literature, although we personally criticise this term (see Harsanyi & Carbon, 2015). One influential explanation for the other-race effect is that reduced perceptual expertise with faces from less familiar morphological groups may result in a comparatively weaker reliance on configural processing (Diamond & Carey, 1986) and greater reliance on feature-based strategies (e.g., Meissner & Brigham, 2001; Tanaka et al., 2004). This account has frequently been invoked to explain the own-race bias observed in face recognition studies (e.g., Lindsay et al., 1991; Meissner & Brigham, 2001). The bias was demonstrated across several study paradigms involving different racial groups (see Meissner & Brigham, 2001). However, findings are mixed, and some studies suggest that own- and other-race faces may be processed configurally to a similar extent (e.g., Horry et al., 2015; Mondloch et al., 2010). Mondloch and colleagues (2010) tested feature-based processing and the processing of second-order relations and found that an own-race bias occurs in both cases. They concluded that even if faces of different morphological groups are processed equally configurally, other factors that favour an own-race bias (e.g., different experiences with other-race faces) remain. Consequently, the precise mechanisms underlying the own-race bias remain debated.

Several theoretical frameworks have attempted to explain the own-race bias. Expertise-based accounts propose that increased contact with faces from a particular morphological group leads to more efficient and more configural face processing (e.g., Hugenberg et al., 2010; Meissner & Brigham, 2001; Wilson et al., 2016). Several studies confirm the assumption that own-race faces are processed in a more configural way, whereas other-race faces are processed in a more featural way (see e.g., DeGutis et al., 2013; Tanaka et al., 2004). However, alternative approaches emphasise representational differences rather than qualitatively distinct perceptual mechanisms. For instance, Valentine's (1991) multidimensional face-space model proposes that greater experience with own-race faces results in denser and more differentiated representational structures in memory. Recent accounts additionally suggest that extensive experience with a morphological group may increase tolerance toward within-person variability and degraded visual conditions (Burton et al., 2016).

Some studies explicitly addressed the role of contact frequency and familiarity regarding face-race effects. Hancock and Rhodes (2008), for example, demonstrated that increased contact with other-race individuals reduced differences between own- and other-race face recognition performances. Similarly, Sangrigoli et al. (2005) showed that Korean adults adopted by Caucasian families demonstrated a recognition advantage for Caucasian faces, suggesting that perceptual expertise can be substantially altered through experience. Interestingly, the age of arrival did not correlate with the size of the other-race effect: even those who arrived in Europe after age 7 showed an advantage for Caucasian faces.

In summary, substantial evidence suggests a close relationship between perceptual expertise and the own-race bias, although the precise underlying mechanisms remain unclear. The Thatcher illusion may provide a particularly useful paradigm for investigating these mechanisms because it allows systematic examination of orientation-dependent changes in face processing. Hahn et al. (2012) showed with a speeded grotesqueness rating task that inverted, Thatcherised faces lead to stronger impairments in configural processing of own- compared to other-race faces. Based on initial electroencephalography (EEG) findings showing that inverted Thatcher faces show smaller N170 amplitudes (Carbon et al., 2005), Hahn et al. (2012) could show a more pronounced modulation for the N170 component (supposed to be modulated by expertise) for Thatcherised own- compared to other-race faces (with smaller amplitudes for own- compared to other-race faces). Interestingly, mean grotesqueness ratings showed the opposite, suggesting other-race faces were equally (or even more) relying on configural processing than own-race faces. However, the study did not report detailed behavioural accuracy or RT analyses, making the interpretation of the findings difficult, as different processes can be assumed for different decision and response qualities.

### The Present Study

The primary aim of the present study was to examine the own-race bias by means of the Thatcher illusion paradigm. This paradigm has robustly revealed disrupted configural processing in inverted faces. Such disruption of configural processing was also documented for other-race faces (e.g., Meissner & Brigham, 2001). Preliminary research combining a Thatcher illusion paradigm with other-race faces has revealed stronger impairments in configural processing of own- compared to other-race faces for inverted Thatcherised items (e.g., Hahn et al., 2012) when analysing Event-Related Potential (ERP) components. Grotesqueness ratings contradicted those results. We adopted the gradual rotation paradigm introduced by Carbon et al. (2007), who initially used this paradigm to analyse the amount of configural face processing in persons with cPA. By using own- versus other-race faces (Thatcherised or not) across seven different orientations ranging from 0° to 180° in 30° steps, we were able to analyse the amount of configural processing in more detail: Since other-race faces are supposed to be processed in a more featural than configural way, we hypothesised that RTs for Thatcherised own-race faces would descriptively follow a more abrupt sigmoid-like response profile across orientations, whereas RTs for Thatcherised other-race faces would show a more gradual and approximately linear increase with orientation, consistent with a comparatively greater reliance on feature-based processing strategies.

## Method

### Participants

A minimum *N* of participants was calculated *a priori* via G*Power (Faul et al., 2007), based on a 2 [face race] × 2 [Thatcherisation] × 7 [orientations] within-participants ANOVA revealing a medium-sized effect of *f* = 0.25, α = 0.05 and a test power 1-β = 0.80. On the basis of an *N* of 23 needed to reveal such an effect for the interaction of the main variables in the repeated-measures ANOVA, we planned to recruit a sample of greater than 28 (20% more participants) to compensate for typical drop-outs and excluded participants due to high outlier rates in speeded reaction tasks. 31 participants (20 female, 11 male) with ages ranging between 19 and 27 years (*M*_age_ = 22.3 years; *SD*_age_ = 2.0) took part. The final sample after excluding participants due to outlier criteria (see below) consisted of 27 participants (18 female, nine male) with ages ranging between 19 and 27 (*M*_age_ = 22.1 years; *SD*_age_ = 1.9). All participants had normal or corrected-to-normal vision (assessed by a standard Snellen eye chart test; Snellen, 1862). All had normal colour vision, assessed by a short version of the Ishihara colour test (Ishihara, 1917). Furthermore, even mild contact with persons of the focused other morphological group (assessed by the contact questionnaire by Hancock & Rhodes, 2008) leads to an exclusion of data in order to prevent too much experience or expertise in the recognition and processing of faces of the focused other morphological group. We used this criterion on which none of the participants had to be excluded.

Participants were undergraduates of the University of Bamberg; they received course credit points for their participation. They had no prior experience with the present task and were naive to the purpose of this experiment. The study was conducted according to the principles expressed in the Declaration of Helsinki and the ethical principles of the German Psychological Society (DFG) and the Association of German Professional Psychologists (BDP). Participants were made aware of their right to withdraw themselves and their data from the study without consequences and without giving reasons. Written informed consent was then given by each participant. The details and the rationale of the study were discussed with every participant on completion of the experiment. The general study design (psychophysical testing) was given ethical approval by the local ethics committee of the University of Bamberg.

### Apparatus

Participants were seated approximately 60 cm in front of a 61.2 cm EIZO ColorEdgeCG245W-LC monitor running at a 1,920 × 1,200-pixel screen resolution with a refresh rate of 60 Hz controlled by a Fujitsu PC. Participants responded on a Cedrus BottonBox (USB Response Pad RB-530; with a delay of under 500 μs for signal output) with two buttons. Participants had to press one button for ‘yes’, the other for ‘no’. Which button corresponded to which reply was varied randomly across participants. Stimuli, trials, and experimental blocks were created with the up-to-date SR Research Experiment Builder^©^ 1.10.1025, ensuring high precision in executing the correct timing of the study (typically ≤1 ms accuracy, also for measuring the RTs).

### Stimuli and Materials

All participants had to complete a questionnaire regarding their contact with people of their own and of the other race tested in the present experiments. The questionnaire used by Hancock and Rhodes (2008) was translated into German and adapted to the morphological groups used in the present study (Caucasian and Black faces).

**Figure 1.**
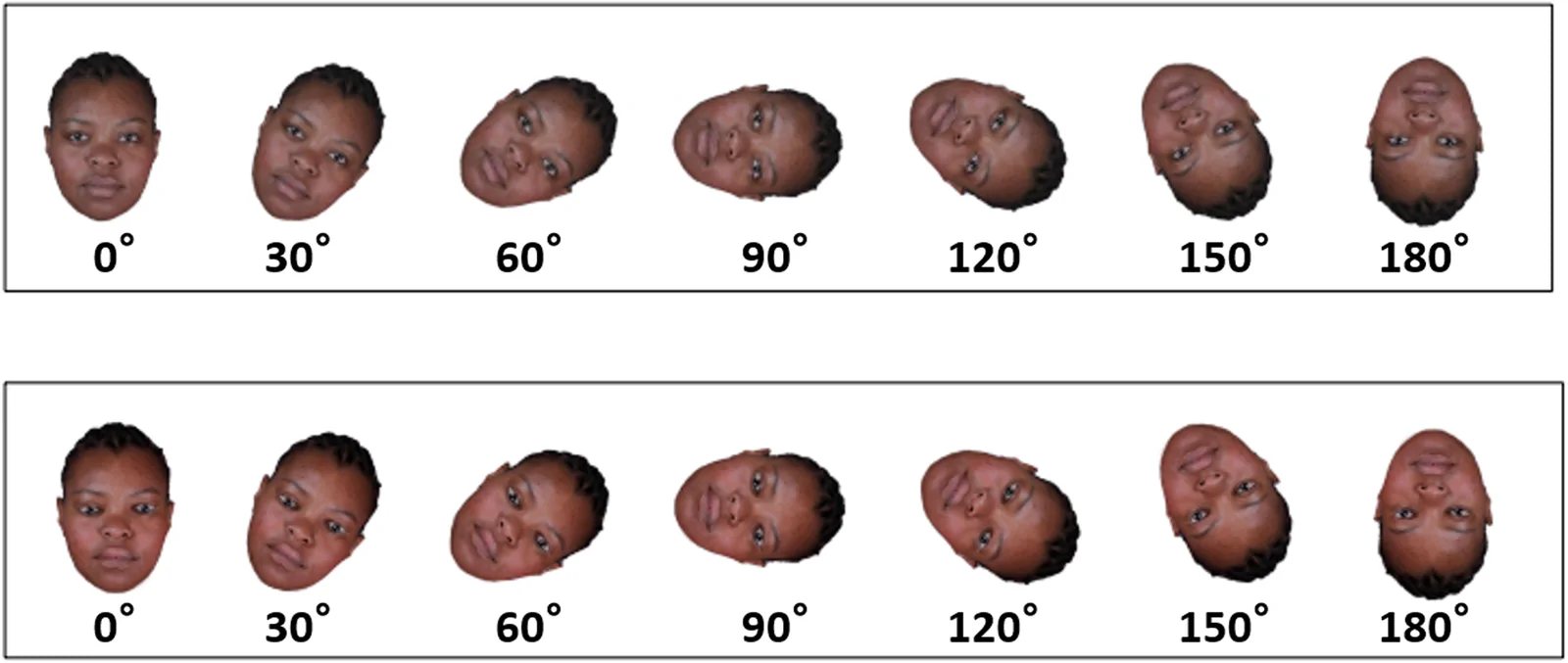
Example stimuli for normal (top row) and Thatcherised (bottom row) other-race faces for the seven different orientations (0°–180°).

Frontal versions of Caucasian faces were taken from the BA-DADA face database (^©^ by CCC at University of Bamberg), and Black face pictures from a database kindly provided to CCC by Professor Colin Tredoux (University of Cape Town, South Africa). Eight Black and eight Caucasian faces (half male, half female) were used. The selection of pictures was based on a neutral facial expression; no prominent hairstyle or facial feature was present with the selected stimuli. Using Adobe Photoshop CS4, the neck and chest were removed from the images, and eyes and mouth were inverted by 180° for the Thatcherisation purpose. Stimuli were approximately 3.5 cm wide and 6.5 cm high (related to the upright orientation). All pictures were rotated clockwise in steps of 30° from 0° (upright) to 180° (inverted), resulting in seven different orientations (see Figure 1 for an example).

### Design and Procedure

Participants first completed four practice trials designed to familiarise them with the task structure, timing, and response requirements, rather than to train the detection of Thatcherised faces. Each trial presented a single face, and participants were asked to discriminate whether it was ‘grotesque’ or not as quickly and accurately as possible. Feedback was provided during practice trials only. After those practice trials, in a two-alternative forced-choice (2AFC) task, 224 faces (2 ‘races’, i.e., morphological groups [Black/Caucasian] × 8 faces × 2 Thatcherisation [yes/no] × 7 orientation [0°, 30°, 60°, 90°, 120°, 150°, 180°]) were presented in random order. Participants had to decide as quickly and as accurately as possible whether the presented face looked ‘grotesque’ or not. Experimental trials were organised in four blocks of 56 trials. Between blocks, participants were free to take a break. Each trial started with an instruction screen for 2,000 ms ‘Is the following face grotesque or not?’ followed by a black fixation cross in the middle of the screen. After a blank screen (500 ms), the actual target face was presented for 1,000 ms or until participants answered using the button box. After 1,000 ms, participants saw a blank screen until they answered. The whole procedure took approximately 30–45 min.

### Data Analysis and Curve Fitting

To descriptively characterise orientation-dependent changes in RTs, curve fitting was applied to grand-mean RTs across the seven rotation levels. Following Carbon et al. (2007), RTs for Thatcherised own-race faces were modelled using a sigmoid Fermi–Dirac function:

$$f(x)=\frac{a}{\exp\;{\left(\frac{x-b}{c}\right)}^{+}1}+d$$

where (a) represents amplitude, (b) the location of the inflection point, (c) the steepness of the transition between the two asymptotes and (d) the upper asymptote, whereas the lower asymptote is given by d + a. The sigmoid function was intended as a descriptive model capturing a hypothesised abrupt transition from predominantly configural to increasingly feature-based processing as facial orientation deviates from upright presentation.

RTs for Thatcherised other-race faces were fitted with a linear function, *f*(x) = mx + b), reflecting a gradual increase across rotations.

Curve fitting was performed using nonlinear least-squares estimation in R with the nlsLM function from the minpack.lm package. Model fit (*R*^2^) was quantified using the coefficient of determination: *R*^2^ = 1 − SSE/SST, where SSE is the residual sum of squares and SST is the total sum of squares. Fits were performed on unweighted grand-mean RT values across the seven rotation levels. No parameter constraints were applied. Convergence was assessed based on successful termination of the nonlinear optimisation algorithm. The sigmoid and linear models were also applied to both conditions for descriptive comparison; however, formal statistical comparison of model fit (e.g., AICc) was performed for transparency. The analysis code is available in the OSF repository (https://osf.io/s9q8n/overview?view_only = a804f2aade604989aa264c4a62e3d786).

## Results

As the main aim was to investigate the own-race bias more closely with the help of the Thatcher illusion task, we focused on analysing the participants’ RTs and correct responses. Trials with RTs faster than 200 ms and higher than 2 SDs above each participant's individual mean RT were qualified as outliers and were consequently excluded from further data analysis. We decided to exclude four participants from further analyses due to an extraordinarily high outlier rate of more than 5%. On average, the data loss due to outliers amounted to 2.7%.

RTs and correct responses were then analysed using a three-factor repeated-measures Analysis of Variance (ANOVA) with the factors race [or morphological group] (Black/Caucasian), Thatcherisation (yes/no) and orientation (0°, 30°, 60°, 90°, 120°, 150°, 180°). Post-hoc pairwise comparisons were Bonferroni-corrected. Bayesian analyses were additionally conducted to assess evidence for or against critical interaction effects. Effect sizes and confidence intervals are reported where appropriate.

### Reaction Times

Because the primary hypothesis concerned potential differences in orientation-dependent processing profiles between own- and other-race faces, we first examined the three-way interaction between Thatcherisation, Race, and Orientation. This interaction did not reach significance, *F*_(6,156)_ = 1.71, *p* = .122, η_p_^2^ = .062, indicating that orientation-dependent RT profiles did not differ reliably between own- and other-race faces across Thatcherised and normal conditions. We then examined lower-order effects. Race and Thatcherisation interacted significantly, *F*_(1,26)_ = 44.89, *p* < .001, η_p_^2^ = .633: For Thatcherised faces, RTs were higher for other-race compared to own-race faces (*p* = .001), whereas for normal faces, RTs were higher for own-race compared to other-race faces (*p* < .001). Additionally, own-race RTs were higher for normal compared to Thatcherised faces (*p* = .006), and other-race RTs were higher for Thatcherised compared to normal faces (*p* < .001). There was no effect of Thatcherisation, *F*_(1,26)_ = 1.18, *p* = .288, η_p_^2^ = .043 or race, *F*_(1,26)_ = 0.69, *p* = .414, η_p_^2^ = .026, but of Orientation, *F*_(6,156)_ = 13.91, *p* < .001, η_p_^2^ = .349, showing increasing RTs with increasing rotation from 0°. Thatcherisation and Orientation did not interact, *F*_(6,156)_ = 1.41, *p* = .216, η_p_^2^ = .051, and Race × Orientation was marginally significant, *F*_(6,156)_ = 1.94, *p* = .078, η_p_^2^ = .069, with higher RTs for other-race faces compared to own-race faces at 90° rotation (*p* = .027). To further evaluate the marginal Race × Orientation interaction in RTs, we complemented the frequentist analysis with a Bayesian calibration of the *p* value. This analysis indicated that the evidence for the interaction was at most anecdotal (BF_10,max_ ≈ 1.85). Thus, the marginal frequentist effect should be interpreted cautiously and does not provide strong support for a reliable Race × Orientation interaction. Means (*M*) and standard deviations (*SD*) for RTs across all conditions are reported in Table 1.

**Table 1.** Descriptive statistics (means and standard deviations) for RT (ms) as a function of Race, Thatcherisation and Orientation.

Orientation	Own-Race Thatcherised *M* (*SD*)	Own-Race Normal *M* (*SD*)	Other-Race Thatcherised *M* (*SD*)	Other-Race Normal *M* (*SD*)
0°	810.4 (188.0)	895.4 (215.8)	899.0 (202.7)	797.1 (156.1)
30°	797.0 (181.6)	913.8 (216.8)	919.6 (194.0)	820.7 (190.6)
60°	823.6 (184.8)	876.8 (188.0)	950.1 (229.2)	805.0 (149.8)
90°	815.0 (181.7)	912.8 (196.8)	977.0 (235.1)	888.1 (214.6)
120°	892.8 (206.5)	934.1 (226.4)	983.5 (224.1)	850.6 (221.4)
150°	937.7 (251.6)	969.7 (283.1)	962.6 (261.5)	923.6 (231.6)
180°	955.7 (234.8)	1,000.8 (254.8)	1,032.3 (240.3)	929.0 (236.0)

*Note:* RT = reaction time in milliseconds. Values are presented as M (means) (SD = standard deviations).

### Curve Fitting

**Figure 2.**
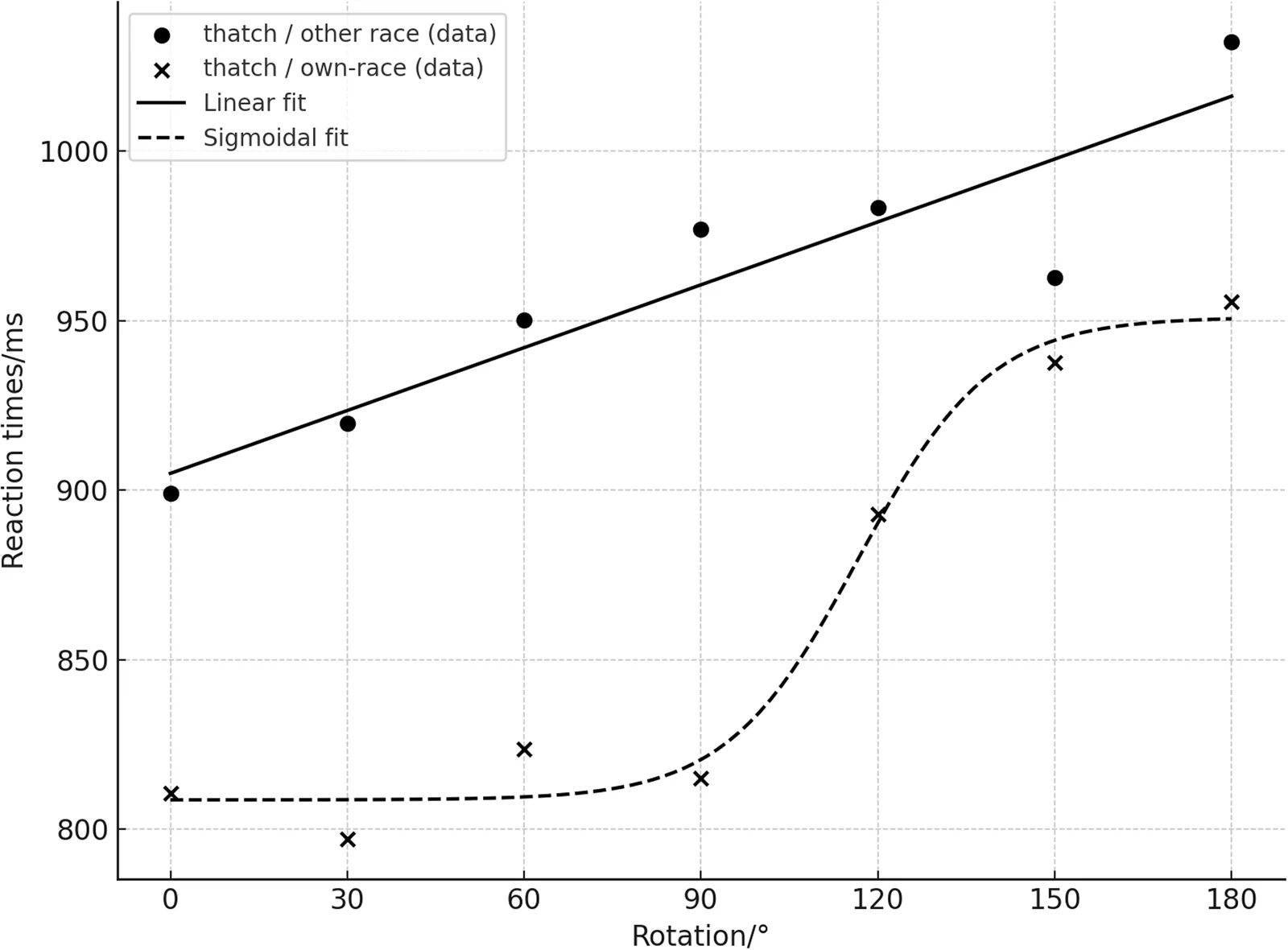
Fitting the averaged RT data for Thatcherised other-race faces with a linear function and Thatcherised own-race faces with a descriptive sigmoid (Fermi–Dirac) function. RT = reaction time.

To provide a descriptive characterisation of the observed response profiles, a Fermi–Dirac function was fitted to the Thatcherised own-race data following Carbon et al. (2007), whereas a linear function was fitted to the Thatcherised other-race data (Figure 2). The distribution of RTs for the Thatcherised other-race condition follows a nearly linear relation (*R*^2^ = .84). In contrast, the increase in RTs with the rotational angle follows a sigmoid function in the Thatcherised own-race condition (*R*^2^ = .98; see Figure 2). For curve fitting, a Fermi–Dirac function $f(x)=\frac{-142.47}{\exp\;\left(\frac{x-4.89}{0.37}\right)+1}+951.05$ was used, following the idea of Carbon et al. (2007), with *a* = −142.47, *b* = 4.89, *c* = 0.37, *d* = 951.05 and as rotation level *x* = 1, 2, 3, 4, 5, 6, and 7 corresponding to 0°, 30°, 60°, 90°, 120°, 150°, and 180° of orientation [rotation in degrees = 30 (*x*–1)]. The estimated lower (d + a) and upper asymptotes (*d*) were 808.6 ms and 951.1 ms, respectively, with the transition point occurring at *x* = 4.89 (corresponding to 116.7° of rotation).

The Fermi–Dirac function provided an excellent descriptive fit for the own-race condition (*R*^2^ = .98), while the linear function provided a good description of the other-race data (*R*^2^ = .84). However, additional linear and Fermi–Dirac models were compared using the corrected Akaike Information Criterion (AICc). Although the more parsimonious linear model was favoured in both conditions, relative support for the Fermi–Dirac model was substantially stronger for own-race faces (ΔAICc = 5.75) than for other-race faces (ΔAICc = 20.83). Detailed results of the curve-fitting procedure, including the estimated Fermi–Dirac model parameters, are provided in the Supplemental Material (Table S1). The fitted functions should therefore be interpreted as descriptive characterisations of the observed response profiles rather than evidence for distinct underlying processing functions.

### Correct Responses

Because our primary hypothesis concerned differences in orientation-dependent performance profiles, we first examined the three-way interaction between Thatcherisation, Race and Orientation. This interaction was significant, *F*_(6,156)_ = 4.85, *p* < .001, η_p_^2^ = .157. For other-race faces, there were significantly more correct responses for normal compared to Thatcherised faces (all *p*s < .006). For own-race faces, this difference was only significant for 0° (*p* = .017). For Thatcherised faces, there were significantly more correct responses for own-race compared to other-race faces (all *p*s < .010). For normal faces, there were more correct responses for other-race compared to own-race faces (*p*s < .05; except for 60° [*p* = .124, *n.s*.] and 180° [*p* = .562, *n.s*.]. Follow-up analyses revealed significant two-way interactions: The interaction of Thatcherisation and Race, *F*_(1,26)_ = 80.70, *p* < .001, η_p_^2^ = .756, showed for Thatcherised faces, higher accuracy for own-race compared to other-race faces (*p* = .001), whereas for normal faces, the reverse was observed (*p* = .007). For other-race faces, correct responses were significantly higher for normal than Thatcherised faces (*p* < .001), but for own-race faces, there was no difference (*p* = .321). The significant interaction of Thatcherisation and Orientation, *F*_(6,156)_ = 7.91, *p* < .001, η_p_^2^ = .233, indicated a decrease in accuracy for Thatcherised faces from 90° onward (*p*s < .04) and for normal faces from 120° onward (*p*s < .03). Race and Orientation also interacted significantly, *F*_(6,156)_ = 7.91, *p* < .001, η_p_^2^ = .233, and showed that own-race faces were more accurate than other-race faces across all orientations (all *p*s < .005) and that accuracy declined for other-race faces from 90° onward (all *p*s < .005) and for own-race faces from 120° onward (*p*s < .007). There was a significant main effect of Thatcherisation, *F*_(1,26)_ = 17.77, *p* < .001, η_p_^2^ = .406, with more correct responses to normal (*M* = 88.1%; *SD* = 18.0) compared to Thatcherised faces (*M* = 72.1%; *SD* = 22.1). There was also a significant effect of race, *F*_(1,26)_ = 42.06, *p* < .001, η_p_^2^ = .618, with more correct responses to own- (*M* = 86.3%; *SD* = 18.1) compared to other-race faces (*M* = 73.8%; *SD* = 22.5). The significant effect of orientation, *F*_(6,156)_ = 49.20, *p* < .001, η_p_^2^ = .654, showed a significant decrease in correct responses from 90° rotation onward (all *p*s < .03). Means and standard deviations for correct responses across all conditions are reported in Table 2.

**Table 2.** Descriptive statistics (means and standard deviations) for correct responses (%) as a function of Race, Thatcherisation and Orientation.

Orientation	Own-Race Thatcherised *M* (*SD*)	Own-Race Normal *M* (*SD*)	Other-Race Thatcherised *M* (*SD*)	Other-Race Normal *M* (*SD*)
0°	97.8 (4.7)	92.8 (9.2)	74.9 (26.3)	98.6 (4.1)
30°	93.5 (11.7)	88.7 (19.5)	79.3 (23.9)	94.1 (17.0)
60°	91.9 (12.5)	89.7 (14.3)	76.7 (23.6)	89.8 (17.9)
90°	82.8 (19.9)	88.6 (13.6)	63.6 (25.2)	87.9 (13.2)
120°	75.6 (21.5)	89.2 (12.5)	56.6 (24.5)	81.2 (16.1)
150°	74.9 (23.1)	81.8 (19.0)	51.7 (24.4)	74.2 (21.8)
180°	72.9 (21.0)	85.2 (15.9)	49.9 (24.6)	72.4 (21.2)

*Note:* Values are percentages of correct responses. Data are presented as M (means) (SD = standard deviations).

Overall, these results highlight orientation- and race-dependent differences in both RT and correct responses, with descriptive curve fitting showing a steeper transition for own-race Thatcherised faces and a more gradual linear increase in RTs for other-race Thatcherised faces.

## Discussion

The present study aimed to examine the own-race bias by using a modified Thatcher illusion paradigm that combined gradual orientation manipulations with own- and other-race faces. Compared with the traditional upright-versus-inverted contrast, this design allowed a more fine-grained analysis of orientation-dependent changes in face processing. Previous work combining a Thatcher illusion paradigm with other-race faces has revealed stronger inversion-related impairments for own- compared to other-race faces in ERP measures (e.g., Hahn et al., 2012). Grotesqueness ratings contradicted those results. However, these results are difficult to interpret as essential measures such as performance and RT measures were not provided by the authors. By adopting the gradual rotation paradigm introduced by Carbon et al. (2007), the present study aimed to characterise orientation-dependent processing profiles in greater detail. Consistent with our hypotheses, descriptive curve fitting suggested differing orientation-dependent response profiles for own- and other-race Thatcherised faces. For own-race faces, RTs descriptively followed a sigmoidal function with a marked increase around 120° rotation, whereas RTs for other-race faces showed a more gradual and approximately linear increase across orientations. Importantly, however, these fitting procedures were intended as descriptive characterisations of the observed response profiles rather than as inferential tests of qualitatively distinct processing modes. Furthermore, the absence of a significant three-way interaction for RTs suggests that interpretations regarding categorical processing differences should be made cautiously. Accuracy data nevertheless supported the notion of orientation-dependent differences between own- and other-race face processing. In particular, Thatcherised other-race faces showed a pronounced decline in performance beginning at lower rotation angles compared with own-race faces. In sum, these findings are broadly consistent with previous studies suggesting that own-race faces are processed with a stronger reliance on configural and/or holistic processing strategies, whereas other-race faces may rely comparatively more strongly on feature-based processing (e.g., Meissner & Brigham, 2001; Tanaka et al., 2004).

The present findings should also be considered in relation to Hahn et al. (2012), who reported stronger inversion-related impairments for own-race than other-race faces using ERP measures. At first glance, their findings may appear to be discrepant with the current behavioural results. However, several methodological differences should be considered, whereas Hahn et al. employed electrophysiological measures that primarily reflect early perceptual and neural processing stages, the present study assessed behavioural responses that integrate perceptual, decisional, and motor components. Furthermore, ERP and behavioural measures may capture partially distinct aspects of configural and holistic processing. Consequently, direct comparisons between the two studies should be interpreted cautiously. Nevertheless, both studies converge in suggesting differential processing of own- and other-race faces as a function of orientation.

The present findings also partially resemble the response profiles reported by Carbon et al. (2007) for individuals with cPA. In that study, participants with cPA showed more gradual, approximately linear RT patterns across orientations, whereas controls exhibited a more abrupt sigmoid-like transition pattern. A superficially similar dissociation was observed in the present study when comparing own- and other-race faces. However, this similarity should be interpreted cautiously. The present findings do not imply that processing of other-race faces in typical observers is equivalent to face processing in cPA more generally. Rather, the similarity appears limited to the orientation-dependent response profiles observed within the Thatcher illusion paradigm. Processes involved in identity matching, familiarity-based recognition or within-person variability processing likely rely on partially distinct mechanisms. Importantly, the present comparison should not be taken to imply that observers processing other-race faces exhibit impairments comparable in magnitude to those observed in cPA more generally. Individuals with cPA typically show pronounced deficits across a broad range of face-processing tasks, including face learning, recognition, and identity matching. In contrast, participants in the present study showed relatively high levels of performance even for other-race faces. Thus, the observed similarity appears restricted to the orientation-dependent response profiles within the Thatcher illusion paradigm and should not be generalised to broader aspects of face-processing ability.

It should also be noted that the present study did not require participants to memorise the faces. Therefore, any suggestion that the observed own-race bias reflects differences in memory representations is tentative. The results are better interpreted in terms of orientation- and feature-dependent processing within the perceptual domain, rather than as evidence for differential memory encoding or retrieval. The present findings should be interpreted within the specific context of the Thatcher illusion paradigm. The task required participants to discriminate between Thatcherised and non-Thatcherised faces and therefore primarily assessed sensitivity to orientation-dependent configurational distortions. Consequently, the present findings should not be generalised to broader aspects of face recognition, such as identity matching, familiarity-based categorisation, face learning or the processing of within-person variability. Previous research suggests that sensitivity to configurational manipulations and successful identity recognition may rely on partially distinct mechanisms and do not necessarily correlate strongly (e.g., Sandford & Bindemann, 2020). Likewise, tasks requiring observers to reconcile highly variable images of the same individual may involve processes that extend beyond those assessed by the present paradigm (e.g., Jenkins et al., 2011). Therefore, although the present findings are broadly consistent with expertise-based accounts of face perception, they should not be interpreted as evidence that increasing reliance on configural processing alone would necessarily improve face-recognition performance more generally.

From a theoretical perspective, the present findings are broadly consistent with expertise-based accounts of the own-race bias. Increased experience with a particular morphological group may facilitate more efficient configural processing and more differentiated face representations (Hugenberg et al., 2010; Meissner & Brigham, 2001; Valentine, 1991). At the same time, recent theoretical perspectives suggest that expertise may additionally increase tolerance toward within-person variability and degraded viewing conditions rather than reflecting a purely qualitative shift between configural and featural processing modes (Burton et al., 2016). Consequently, the present findings should not be interpreted as evidence for a complete absence of configural processing for other-race faces but rather as reflecting reduced reliance on configural/holistic strategies within the present paradigm.

The present study further highlights the usefulness of gradual rotation paradigms for investigating orientation-dependent changes in face processing. Unlike simple upright-versus-inverted comparisons, gradual rotation allows identification of the orientations at which performance begins to deteriorate and provides a more nuanced characterisation of face-processing dynamics. Such approaches may therefore contribute to a more detailed understanding of the perceptual mechanisms underlying the own-race bias.

Importantly, the present findings should also be interpreted in light of ongoing debates regarding the interpretation of inversion effects and the Thatcher illusion. Picture-plane inversion likely affects multiple aspects of face processing simultaneously, including first-order relations, second-order relations, and holistic/configural processing (Maurer et al., 2002). Consequently, inversion-related impairments should not be interpreted as selectively reflecting isolated configural mechanisms. Similarly, the extent to which the Thatcher illusion directly indexes holistic processing remains debated (e.g., McKone & Yovel, 2009; Psalta et al., 2014).

### Limitations

Several limitations should be acknowledged. First, although we controlled for contact with other-race individuals, cultural and individual differences in face-processing expertise cannot be entirely ruled out. Second, the study focused exclusively on Caucasian and Black faces, restricting conclusions to these two morphological groups. Third, the curve fitting procedures were performed on grand-mean data and should therefore be interpreted descriptively. Future studies may benefit from participant-level modelling approaches and formal model-comparison procedures. Furthermore, future research should include a broader range of morphological groups and combine behavioural data with neurophysiological measures (e.g., EEG, functional Magnetic Resonance Imaging [fMRI]) to identify the neural mechanisms underlying orientation-dependent processing differences. Training studies investigating whether extensive exposure to other-race faces can shift processing strategies, as suggested by studies on perceptual expertise and adoption-related reversal of the own-race effect (e.g., Sangrigoli et al., 2005), would also be highly interesting.

Taken together, the present findings suggest that own- and other-race faces differ in their orientation-dependent response profiles within the Thatcher illusion paradigm. Whereas own-race faces showed a more abrupt sigmoid-like transition pattern, other-race faces showed a more gradual response profile across orientations. These findings are broadly consistent with the notion that perceptual expertise influences the balance between configural/holistic and feature-based processing strategies in face perception.

## Supplemental Material

sj-docx-1-ipe-10.1177_20416695261480594 - Supplemental material for Impaired configural processing for other-race faces revealed by the Thatcher illusion Supplemental material, sj-docx-1-ipe-10.1177_20416695261480594 for Impaired configural processing for other-race faces revealed by the Thatcher illusion by Sandra Utz and Claus-Christian Carbon in i-Perception

## Notes

1. We recognise that the term ‘other-race effect’ is problematic, as it invokes socially constructed racial categories rather than more precise descriptors such as morphological variation or population-specific familiarity. Nevertheless, this terminology remains the dominant convention in perceptual and cognitive science. We use it here for continuity with prior research but emphasize the need for future work to adopt terminology that better reflects the underlying perceptual and experiential factors involved.
