# Supplementary material for: Impaired configural processing for other-race faces revealed by the Thatcher illusion
Source: Iperception. 2026 Sep 7;17(5):20416695261480594. doi: 10.1177/20416695261480594 (PMC13554658; doi:10.1177/20416695261480594)
Supplement: Supplementary file 1 [file sj-docx-1-ipe-10.1177_20416695261480594.docx]

**Table S1**

*Relative fit of linear and Fermi–Dirac models for Thatcherised own- and other-race faces, assessed using corrected Akaike Information Criterion (AICc).*

| Race | Model | Number of parameters (*k*) | AICc | ΔAICc | Akaike weight |
| --- | --- | --- | --- | --- | --- |
| Own-race faces | Linear model | 2 | 51.34 | 0.00 | 0.947 |
|  | Fermi-Dirac model | 4 | 57.09 | 5.75 | 0.053 |
| Other-race faces | Linear model | 2 | 46.20 | 0.00 | 1.000 |
|  | Fermi-Dirac model | 4 | 67.03 | 20.83 | < 0.001 |
